# Ensembl 2022

**DOI:** 10.1093/nar/gkab1049

**Published:** 2021-11-17

**Authors:** Fiona Cunningham, James E Allen, Jamie Allen, Jorge Alvarez-Jarreta, M Ridwan Amode, Irina M Armean, Olanrewaju Austine-Orimoloye, Andrey G Azov, If Barnes, Ruth Bennett, Andrew Berry, Jyothish Bhai, Alexandra Bignell, Konstantinos Billis, Sanjay Boddu, Lucy Brooks, Mehrnaz Charkhchi, Carla Cummins, Luca Da Rin Fioretto, Claire Davidson, Kamalkumar Dodiya, Sarah Donaldson, Bilal El Houdaigui, Tamara El Naboulsi, Reham Fatima, Carlos Garcia Giron, Thiago Genez, Jose Gonzalez Martinez, Cristina Guijarro-Clarke, Arthur Gymer, Matthew Hardy, Zoe Hollis, Thibaut Hourlier, Toby Hunt, Thomas Juettemann, Vinay Kaikala, Mike Kay, Ilias Lavidas, Tuan Le, Diana Lemos, José Carlos Marugán, Shamika Mohanan, Aleena Mushtaq, Marc Naven, Denye N Ogeh, Anne Parker, Andrew Parton, Malcolm Perry, Ivana Piližota, Irina Prosovetskaia, Manoj Pandian Sakthivel, Ahamed Imran Abdul Salam, Bianca M Schmitt, Helen Schuilenburg, Dan Sheppard, José G Pérez-Silva, William Stark, Emily Steed, Kyösti Sutinen, Ranjit Sukumaran, Dulika Sumathipala, Marie-Marthe Suner, Michal Szpak, Anja Thormann, Francesca Floriana Tricomi, David Urbina-Gómez, Andres Veidenberg, Thomas A Walsh, Brandon Walts, Natalie Willhoft, Andrea Winterbottom, Elizabeth Wass, Marc Chakiachvili, Bethany Flint, Adam Frankish, Stefano Giorgetti, Leanne Haggerty, Sarah E Hunt, Garth R IIsley, Jane E Loveland, Fergal J Martin, Benjamin Moore, Jonathan M Mudge, Matthieu Muffato, Emily Perry, Magali Ruffier, John Tate, David Thybert, Stephen J Trevanion, Sarah Dyer, Peter W Harrison, Kevin L Howe, Andrew D Yates, Daniel R Zerbino, Paul Flicek

**Affiliations:** European Molecular Biology Laboratory, European Bioinformatics Institute, Wellcome Genome Campus, Hinxton, Cambridge CB10 1SD, UK; European Molecular Biology Laboratory, European Bioinformatics Institute, Wellcome Genome Campus, Hinxton, Cambridge CB10 1SD, UK; European Molecular Biology Laboratory, European Bioinformatics Institute, Wellcome Genome Campus, Hinxton, Cambridge CB10 1SD, UK; European Molecular Biology Laboratory, European Bioinformatics Institute, Wellcome Genome Campus, Hinxton, Cambridge CB10 1SD, UK; European Molecular Biology Laboratory, European Bioinformatics Institute, Wellcome Genome Campus, Hinxton, Cambridge CB10 1SD, UK; European Molecular Biology Laboratory, European Bioinformatics Institute, Wellcome Genome Campus, Hinxton, Cambridge CB10 1SD, UK; European Molecular Biology Laboratory, European Bioinformatics Institute, Wellcome Genome Campus, Hinxton, Cambridge CB10 1SD, UK; European Molecular Biology Laboratory, European Bioinformatics Institute, Wellcome Genome Campus, Hinxton, Cambridge CB10 1SD, UK; European Molecular Biology Laboratory, European Bioinformatics Institute, Wellcome Genome Campus, Hinxton, Cambridge CB10 1SD, UK; European Molecular Biology Laboratory, European Bioinformatics Institute, Wellcome Genome Campus, Hinxton, Cambridge CB10 1SD, UK; European Molecular Biology Laboratory, European Bioinformatics Institute, Wellcome Genome Campus, Hinxton, Cambridge CB10 1SD, UK; European Molecular Biology Laboratory, European Bioinformatics Institute, Wellcome Genome Campus, Hinxton, Cambridge CB10 1SD, UK; European Molecular Biology Laboratory, European Bioinformatics Institute, Wellcome Genome Campus, Hinxton, Cambridge CB10 1SD, UK; European Molecular Biology Laboratory, European Bioinformatics Institute, Wellcome Genome Campus, Hinxton, Cambridge CB10 1SD, UK; European Molecular Biology Laboratory, European Bioinformatics Institute, Wellcome Genome Campus, Hinxton, Cambridge CB10 1SD, UK; European Molecular Biology Laboratory, European Bioinformatics Institute, Wellcome Genome Campus, Hinxton, Cambridge CB10 1SD, UK; European Molecular Biology Laboratory, European Bioinformatics Institute, Wellcome Genome Campus, Hinxton, Cambridge CB10 1SD, UK; European Molecular Biology Laboratory, European Bioinformatics Institute, Wellcome Genome Campus, Hinxton, Cambridge CB10 1SD, UK; European Molecular Biology Laboratory, European Bioinformatics Institute, Wellcome Genome Campus, Hinxton, Cambridge CB10 1SD, UK; European Molecular Biology Laboratory, European Bioinformatics Institute, Wellcome Genome Campus, Hinxton, Cambridge CB10 1SD, UK; European Molecular Biology Laboratory, European Bioinformatics Institute, Wellcome Genome Campus, Hinxton, Cambridge CB10 1SD, UK; European Molecular Biology Laboratory, European Bioinformatics Institute, Wellcome Genome Campus, Hinxton, Cambridge CB10 1SD, UK; European Molecular Biology Laboratory, European Bioinformatics Institute, Wellcome Genome Campus, Hinxton, Cambridge CB10 1SD, UK; European Molecular Biology Laboratory, European Bioinformatics Institute, Wellcome Genome Campus, Hinxton, Cambridge CB10 1SD, UK; European Molecular Biology Laboratory, European Bioinformatics Institute, Wellcome Genome Campus, Hinxton, Cambridge CB10 1SD, UK; European Molecular Biology Laboratory, European Bioinformatics Institute, Wellcome Genome Campus, Hinxton, Cambridge CB10 1SD, UK; European Molecular Biology Laboratory, European Bioinformatics Institute, Wellcome Genome Campus, Hinxton, Cambridge CB10 1SD, UK; European Molecular Biology Laboratory, European Bioinformatics Institute, Wellcome Genome Campus, Hinxton, Cambridge CB10 1SD, UK; European Molecular Biology Laboratory, European Bioinformatics Institute, Wellcome Genome Campus, Hinxton, Cambridge CB10 1SD, UK; European Molecular Biology Laboratory, European Bioinformatics Institute, Wellcome Genome Campus, Hinxton, Cambridge CB10 1SD, UK; European Molecular Biology Laboratory, European Bioinformatics Institute, Wellcome Genome Campus, Hinxton, Cambridge CB10 1SD, UK; European Molecular Biology Laboratory, European Bioinformatics Institute, Wellcome Genome Campus, Hinxton, Cambridge CB10 1SD, UK; European Molecular Biology Laboratory, European Bioinformatics Institute, Wellcome Genome Campus, Hinxton, Cambridge CB10 1SD, UK; European Molecular Biology Laboratory, European Bioinformatics Institute, Wellcome Genome Campus, Hinxton, Cambridge CB10 1SD, UK; European Molecular Biology Laboratory, European Bioinformatics Institute, Wellcome Genome Campus, Hinxton, Cambridge CB10 1SD, UK; European Molecular Biology Laboratory, European Bioinformatics Institute, Wellcome Genome Campus, Hinxton, Cambridge CB10 1SD, UK; European Molecular Biology Laboratory, European Bioinformatics Institute, Wellcome Genome Campus, Hinxton, Cambridge CB10 1SD, UK; European Molecular Biology Laboratory, European Bioinformatics Institute, Wellcome Genome Campus, Hinxton, Cambridge CB10 1SD, UK; European Molecular Biology Laboratory, European Bioinformatics Institute, Wellcome Genome Campus, Hinxton, Cambridge CB10 1SD, UK; European Molecular Biology Laboratory, European Bioinformatics Institute, Wellcome Genome Campus, Hinxton, Cambridge CB10 1SD, UK; European Molecular Biology Laboratory, European Bioinformatics Institute, Wellcome Genome Campus, Hinxton, Cambridge CB10 1SD, UK; European Molecular Biology Laboratory, European Bioinformatics Institute, Wellcome Genome Campus, Hinxton, Cambridge CB10 1SD, UK; European Molecular Biology Laboratory, European Bioinformatics Institute, Wellcome Genome Campus, Hinxton, Cambridge CB10 1SD, UK; European Molecular Biology Laboratory, European Bioinformatics Institute, Wellcome Genome Campus, Hinxton, Cambridge CB10 1SD, UK; European Molecular Biology Laboratory, European Bioinformatics Institute, Wellcome Genome Campus, Hinxton, Cambridge CB10 1SD, UK; European Molecular Biology Laboratory, European Bioinformatics Institute, Wellcome Genome Campus, Hinxton, Cambridge CB10 1SD, UK; European Molecular Biology Laboratory, European Bioinformatics Institute, Wellcome Genome Campus, Hinxton, Cambridge CB10 1SD, UK; European Molecular Biology Laboratory, European Bioinformatics Institute, Wellcome Genome Campus, Hinxton, Cambridge CB10 1SD, UK; European Molecular Biology Laboratory, European Bioinformatics Institute, Wellcome Genome Campus, Hinxton, Cambridge CB10 1SD, UK; European Molecular Biology Laboratory, European Bioinformatics Institute, Wellcome Genome Campus, Hinxton, Cambridge CB10 1SD, UK; European Molecular Biology Laboratory, European Bioinformatics Institute, Wellcome Genome Campus, Hinxton, Cambridge CB10 1SD, UK; European Molecular Biology Laboratory, European Bioinformatics Institute, Wellcome Genome Campus, Hinxton, Cambridge CB10 1SD, UK; European Molecular Biology Laboratory, European Bioinformatics Institute, Wellcome Genome Campus, Hinxton, Cambridge CB10 1SD, UK; European Molecular Biology Laboratory, European Bioinformatics Institute, Wellcome Genome Campus, Hinxton, Cambridge CB10 1SD, UK; European Molecular Biology Laboratory, European Bioinformatics Institute, Wellcome Genome Campus, Hinxton, Cambridge CB10 1SD, UK; European Molecular Biology Laboratory, European Bioinformatics Institute, Wellcome Genome Campus, Hinxton, Cambridge CB10 1SD, UK; European Molecular Biology Laboratory, European Bioinformatics Institute, Wellcome Genome Campus, Hinxton, Cambridge CB10 1SD, UK; European Molecular Biology Laboratory, European Bioinformatics Institute, Wellcome Genome Campus, Hinxton, Cambridge CB10 1SD, UK; European Molecular Biology Laboratory, European Bioinformatics Institute, Wellcome Genome Campus, Hinxton, Cambridge CB10 1SD, UK; European Molecular Biology Laboratory, European Bioinformatics Institute, Wellcome Genome Campus, Hinxton, Cambridge CB10 1SD, UK; European Molecular Biology Laboratory, European Bioinformatics Institute, Wellcome Genome Campus, Hinxton, Cambridge CB10 1SD, UK; European Molecular Biology Laboratory, European Bioinformatics Institute, Wellcome Genome Campus, Hinxton, Cambridge CB10 1SD, UK; European Molecular Biology Laboratory, European Bioinformatics Institute, Wellcome Genome Campus, Hinxton, Cambridge CB10 1SD, UK; European Molecular Biology Laboratory, European Bioinformatics Institute, Wellcome Genome Campus, Hinxton, Cambridge CB10 1SD, UK; European Molecular Biology Laboratory, European Bioinformatics Institute, Wellcome Genome Campus, Hinxton, Cambridge CB10 1SD, UK; European Molecular Biology Laboratory, European Bioinformatics Institute, Wellcome Genome Campus, Hinxton, Cambridge CB10 1SD, UK; European Molecular Biology Laboratory, European Bioinformatics Institute, Wellcome Genome Campus, Hinxton, Cambridge CB10 1SD, UK; European Molecular Biology Laboratory, European Bioinformatics Institute, Wellcome Genome Campus, Hinxton, Cambridge CB10 1SD, UK; European Molecular Biology Laboratory, European Bioinformatics Institute, Wellcome Genome Campus, Hinxton, Cambridge CB10 1SD, UK; European Molecular Biology Laboratory, European Bioinformatics Institute, Wellcome Genome Campus, Hinxton, Cambridge CB10 1SD, UK; European Molecular Biology Laboratory, European Bioinformatics Institute, Wellcome Genome Campus, Hinxton, Cambridge CB10 1SD, UK; European Molecular Biology Laboratory, European Bioinformatics Institute, Wellcome Genome Campus, Hinxton, Cambridge CB10 1SD, UK; European Molecular Biology Laboratory, European Bioinformatics Institute, Wellcome Genome Campus, Hinxton, Cambridge CB10 1SD, UK; European Molecular Biology Laboratory, European Bioinformatics Institute, Wellcome Genome Campus, Hinxton, Cambridge CB10 1SD, UK; European Molecular Biology Laboratory, European Bioinformatics Institute, Wellcome Genome Campus, Hinxton, Cambridge CB10 1SD, UK; European Molecular Biology Laboratory, European Bioinformatics Institute, Wellcome Genome Campus, Hinxton, Cambridge CB10 1SD, UK; European Molecular Biology Laboratory, European Bioinformatics Institute, Wellcome Genome Campus, Hinxton, Cambridge CB10 1SD, UK; European Molecular Biology Laboratory, European Bioinformatics Institute, Wellcome Genome Campus, Hinxton, Cambridge CB10 1SD, UK; European Molecular Biology Laboratory, European Bioinformatics Institute, Wellcome Genome Campus, Hinxton, Cambridge CB10 1SD, UK; European Molecular Biology Laboratory, European Bioinformatics Institute, Wellcome Genome Campus, Hinxton, Cambridge CB10 1SD, UK; European Molecular Biology Laboratory, European Bioinformatics Institute, Wellcome Genome Campus, Hinxton, Cambridge CB10 1SD, UK; European Molecular Biology Laboratory, European Bioinformatics Institute, Wellcome Genome Campus, Hinxton, Cambridge CB10 1SD, UK; European Molecular Biology Laboratory, European Bioinformatics Institute, Wellcome Genome Campus, Hinxton, Cambridge CB10 1SD, UK; European Molecular Biology Laboratory, European Bioinformatics Institute, Wellcome Genome Campus, Hinxton, Cambridge CB10 1SD, UK; European Molecular Biology Laboratory, European Bioinformatics Institute, Wellcome Genome Campus, Hinxton, Cambridge CB10 1SD, UK; European Molecular Biology Laboratory, European Bioinformatics Institute, Wellcome Genome Campus, Hinxton, Cambridge CB10 1SD, UK; European Molecular Biology Laboratory, European Bioinformatics Institute, Wellcome Genome Campus, Hinxton, Cambridge CB10 1SD, UK; European Molecular Biology Laboratory, European Bioinformatics Institute, Wellcome Genome Campus, Hinxton, Cambridge CB10 1SD, UK; European Molecular Biology Laboratory, European Bioinformatics Institute, Wellcome Genome Campus, Hinxton, Cambridge CB10 1SD, UK; European Molecular Biology Laboratory, European Bioinformatics Institute, Wellcome Genome Campus, Hinxton, Cambridge CB10 1SD, UK; European Molecular Biology Laboratory, European Bioinformatics Institute, Wellcome Genome Campus, Hinxton, Cambridge CB10 1SD, UK; European Molecular Biology Laboratory, European Bioinformatics Institute, Wellcome Genome Campus, Hinxton, Cambridge CB10 1SD, UK; European Molecular Biology Laboratory, European Bioinformatics Institute, Wellcome Genome Campus, Hinxton, Cambridge CB10 1SD, UK; European Molecular Biology Laboratory, European Bioinformatics Institute, Wellcome Genome Campus, Hinxton, Cambridge CB10 1SD, UK; European Molecular Biology Laboratory, European Bioinformatics Institute, Wellcome Genome Campus, Hinxton, Cambridge CB10 1SD, UK

## Abstract

Ensembl (https://www.ensembl.org) is unique in its flexible infrastructure for access to genomic data and annotation. It has been designed to efficiently deliver annotation at scale for all eukaryotic life, and it also provides deep comprehensive annotation for key species. Genomes representing a greater diversity of species are increasingly being sequenced. In response, we have focussed our recent efforts on expediting the annotation of new assemblies. Here, we report the release of the greatest annual number of newly annotated genomes in the history of Ensembl via our dedicated Ensembl Rapid Release platform (http://rapid.ensembl.org). We have also developed a new method to generate comparative analyses at scale for these assemblies and, for the first time, we have annotated non-vertebrate eukaryotes. Meanwhile, we continually improve, extend and update the annotation for our high-value reference vertebrate genomes and report the details here. We have a range of specific software tools for specific tasks, such as the Ensembl Variant Effect Predictor (VEP) and the newly developed interface for the Variant Recoder. All Ensembl data, software and tools are freely available for download and are accessible programmatically.

## INTRODUCTION

The Ensembl project develops infrastructure to deliver reference data for genome interpretation for any species. We annotate genome assemblies from public archives, with genes, regulatory regions, variants and comparative data to provide a foundation for scientific research and genome interpretation. We release our high-value reference vertebrate species via the full Ensembl website (https://www.ensembl.org) approximately every 2–3 months. These data are programmatically available through our Application Programming Interfaces (API) and also download files. They are integrated with a suite of Ensembl tools, most notably the Ensembl Variant Effect predictor (VEP) (1), which determines the effect of user-supplied variants on genes, transcripts, and protein sequence, as well as regulatory regions.

In parallel, we created a Rapid Release platform (https://rapid.ensembl.org/) (2) dedicated to release newly sequenced genomes from biodiversity projects at scale. We annotate these species with core information (transcripts, proteins and comparative annotation) as well as provide BLAST functionality and download files. However, we do not yet provide programmatic access or support for other Ensembl tools on Rapid Release. This site is updated every 2 weeks allowing us to release these new genomes at scale and make them available more rapidly.

## OVERVIEW

### Rapid release of genome annotation

This year we have focussed on delivering accurate eukaryotic genome annotation at scale in support of significantly greater biodiversity in genomics. We have transformed our transcript and comparative annotation methods to expedite the efforts of the recent expansion in genomes sequenced. Here, we announce the release of 202 new genomes—more than in any previous year—via our Rapid Release platform (https://rapid.ensembl.org/). Also newly added to Rapid Release this year are our comparative analyses which infer the closest gene homologue. We have specifically developed these to be generated at scale for new assemblies on Rapid Release. We have additionally improved our multiple alignments to keep pace with a quick dissemination of data.

We discuss our first results of annotation in non-vertebrate eukaryotes and our initial ground-up annotation of haplotypes. We report on the extensive collection of repeat libraries we have built that are essential for aligning cross-species protein data for transcript annotation and to generate whole genome alignments.

### Ensembl.org

Alongside this increased breadth of species data, we report updates to the substantial detailed annotation for a growing number of species on the integrated, full Ensembl website (https://www.ensembl.org). For mouse, we moved our annotation to a new single haplotype assembly and updated the regulatory annotation. We generated a new multiple genome alignment and protein trees specifically for mouse strains. For human, we continue our focus on annotation of COVID-19 susceptibility genes, released as part of the Ensembl/GENCODE gene set. We improved our regulatory annotation for the human Y chromosome. We significantly expanded the human allele frequency data and have extended links to information on variants from the literature. We completed our first Matched Annotation from NCBI and EMBL-EBI (MANE) Select set for clinical genes. We added our first release of the MANE Plus Clinical set. MANE transcripts have also been added to the output of the Ensembl VEP.

### Tools, a new website and training

We report the release of an interface to our Ensembl Variant Recoder tool (https://www.ensembl.org/Multi/Tools/VR). This helps match variants that are named differently across the literature and other resources, and works in any species. We detail the extensions and improvements to our VEP annotations, which include predictions from other popular tools.

Furthermore, we describe the extensive work towards a Minimal Acceptable Product (MAP) for our new website http://2020.ensembl.org. This site can now support basic workflows, has a search engine and has sequence download capabilities. Finally, we report on the successes of our global virtual training programme, including open-registration short courses, delivered during the global pandemic.

## EFFICIENT ANNOTATION AT SCALE FOR ALL EUKARYOTIC LIFE

### Improving genome annotation and release

The past twelve months have seen an influx of high-quality genome assemblies from large scale sequencing projects. These projects, including the Darwin Tree of Life (DToL) project, the Vertebrate Genomes Project (VGP) (3) and the Earth BioGenome Project (4), aim to sequence all eukaryotic life. We created project specific pages for DToL and the VGP. These are dedicated landing pages to quickly view, and access data related to the project. Project pages can be found at https://projects.ensembl.org/.

We have revolutionised our annotation methods and release process to support the scale of data generated by modern ambitious sequencing projects. Our annotations on new assemblies are released on a 2-week cycle via our Rapid Release platform http://rapid.ensembl.org and is our primary mechanism for deploying new annotations quickly. The Rapid Release infrastructure of the site, based on Ensembl code, is designed to be more lightweight and responsive than the full, integrated Ensembl website, and supports essential genome browsing functionality, data download and BLAST capabilities. This allows us to release large volumes of new annotations, as expected from DToL, in a timely way. These annotations include gene sets, protein annotation and repeat masking. We have introduced a completely new workflow that has significantly shortened our release process. We have had to focus on scaling and process optimisation for a fast deployment to keep pace with the assemblies released. As a result, we have been able to annotate and release over 202 genomes since launching Rapid Release in June 2020. We have broadened the application of our annotation outside of vertebrates to develop a new system for annotation of non-vertebrate genomes. Using this, we’ve released 28 non-vertebrate genome annotations, focusing on the Lepidoptera, with full annotations of 14 primary and 14 alternative haplotypes. Alternative haplotypes that are close to the expected genome length have been annotated from the ground up, with the same data and methodology as the corresponding primary haplotype. In creating an efficient annotation system, we have incorporated additional software to maximise the value of transcriptomic data including STAR (5) for short read alignments, as well as Scallop (6) and Stringtie2 (7) for transcript reconstruction.

### Scaling comparative analysis

**Figure 1.**
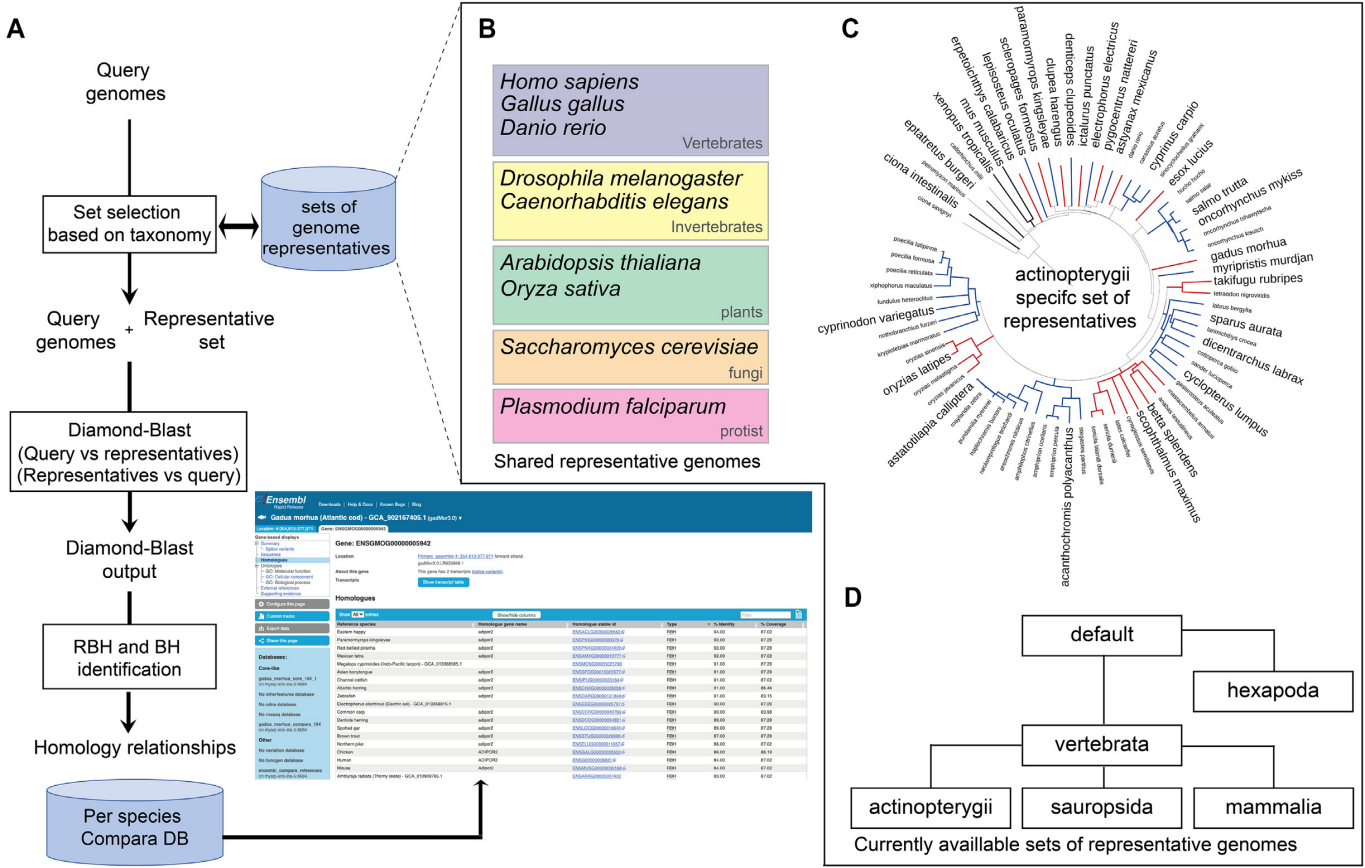
Homology annotation pipeline. (**A**) For each query genome, the pipeline starts with the identification of the correct genome representative set based on the query genome taxonomy. Then the query genome and the representative genomes are compared using Diamond-Blast. The Diamond-Blast output is analysed to identify reciprocal best hit (RBH) or best hit (BH) if no RBH is found against the query genome or representative genomes. The RBH and BH homology relationships are then stored in a per species Compara database. These data are then displayed in the homology view of the Rapid Release platform. (**B**) List of representative genomes shared by all the representative sets. (**C**) The set of representative genomes used for the bony fish (actinopterygii) are here in larger font. Their selection has been based on community usage and annotation quality. The red and blue branches define clusters of actinopterygii subclades identified by their branch length. At least one representative per subclade has been selected. (**D**) The sets of representative genomes that are currently available in Ensembl.

For a comparative analysis of genes for all new genomes on Rapid Release, we built a novel pipeline to infer the closest homologue for any species (Figure 1). We use a new strategy that compares each query genome to a set of 39 representative genomes. The comparison, based on Diamond (8), identifies the reciprocal best hits or the best hit (when no reciprocal best hit is available) to each representative genome to infer the closest homologue. Currently we have developed six representative sets, five defined for the following phyla: vertebrata, mammalia, actinopterygii, sauropsida (including aves and reptilia), hexapoda. Each representative set contains 39 genomes chosen to maximise diversity in a given clade and selected for functional annotation quality and community usage. The six representative sets share nine reference genomes, which are spread across the eukaryotic tree of life. The shared genomes were selected for their importance as model organisms and for their quality of annotation. In addition to the representative sets for specific phyla listed above, we have defined a default representative set. This set is used when no corresponding representative set is defined for a query genome. The default representative set is an extension of the shared reference genome set and includes mostly important model organisms found in each division of the eukaryotic tree of life. New representative sets will be developed as new clades become well represented. In future, we plan to improve the resolution of our homology inference strategy. By integrating leading scientific methods in the field for gene tree inference, we aim to distinguish between orthologues and paralogues. We also plan to add pairwise genome alignments.

We have deployed the Cactus (9) multiple genome aligner software, which is able to compute high quality multiple genome alignments with thousands of genomes in linear time. Moreover, it enables the addition, removal or update of assemblies into an existing alignment. This reduces the amount of process time required to add to existing alignments. Our first large multiple genome alignment built using Cactus consisting of 88 lepidoptera genomes is now available for download in the HAL format from Rapid Release.

### Efficient genome repeat masking at scale

Masking repeats is an important step for aligning cross-species protein data or running whole genome alignments. For non-vertebrate annotations, where there are fewer available repeat libraries, we have used Red (10) for fast masking of the genome for gene annotation and whole genome alignments. We built an extensive collection of repeat libraries via RepeatModeler (11) to identify and classify repeat regions in greater detail. We generated libraries for 1736 genome assemblies, representing 1084 different species. A recent import of 266,740 families from 336 of our libraries into Dfam (12) has resulted in a 40-fold increase in families. At present, most of the libraries represent vertebrate species, however we plan to focus on generating non-vertebrate libraries in the year ahead. The libraries will be used to help classify non-vertebrate repeats, and can be found on the EMBL-EBI ftp site (http://ftp.ebi.ac.uk/pub/databases/ensembl/repeats/unfiltered_repeatmodeler/species/).

## COMPREHENSIVE GENOME ANNOTATION FOR REFERENCE VERTEBRATE SPECIES

We continue to improve and renew the transcript, comparative, regulatory and variation annotation for reference species supported on the full Ensembl website. The data are also available programmatically via our Perl and REST (13) application programming interfaces (APIs), via the BioMart data mining tool and via download files. Notable developments and updates for our reference vertebrate species are described in the following paragraphs.

### New mouse assembly annotation

The mouse genome has been upgraded to the latest assembly, GRCm39, which is a single haplotype with no alternative loci. We transferred all manual annotation to the new assembly, and we corrected any issues. In particular, the improved assembly meant we could fully resolve 34 partial genes, add 13 genes, drop 8 genes as GRCm38 only, and merge 26 genes. We also updated our regulatory build to the latest assembly. For this we processed 78 epigenomes from ENCODE (14) to identify 364,670 putative regulatory elements. We also generated a mouse strain-specific protein tree using our TreeBest-based protein tree pipeline (15).

### Improvements to human annotation including MANE transcripts

For human we have released four updates to our Ensembl/GENCODE reference transcript annotation, created as part of the GENCODE consortium (16). These include continued prioritisation to annotate genes implicated in SARS-CoV2 infection and host response, and COVID-19 disease. As a result, we have now updated over 6200 transcripts, available from Ensembl release 103 (February 2021) onwards.

We released our first set of MANE Select transcripts for all clinical genes that have a confirmed association with human disease including those in the ACMG Secondary Findings genes (17) (Ensembl release 104, May 2021). A MANE Select transcript is a single identical transcript per protein-coding locus that is in both the Ensembl/GENCODE set and NCBI’s RefSeq set (18). Work in this collaboration was encouraged by the results of our recently published survey (19). The latest release of MANE Select (v0.95, available via ftp from July 2021, and in Ensembl release 105) covers 97% of human protein-coding genes. As part of this collaboration, we have introduced ‘MANE Plus Clinical’ transcripts to annotate additional transcripts per locus, when necessary, to support clinical variant reporting of pathogenic variants (from Ensembl release 103, February 2021). This limited set of 56 transcripts are not covered by the MANE Select. We are working on finalising the scope of our first genome-wide release of MANE Select towards the end of 2021. This will exclude, for example genes that are: polymorphic pseudogenes, in areas of the genome with errors, annotated on patches or where both parties cannot agree that the gene is protein coding.

### Annotation updates in other reference vertebrates

Major annotation updates to other key reference species included anole lizard, crab-eating macaque, dog, rat and Tasmanian devil. For dog, we updated the reference assembly to Labrador retriever (ROS_Cfam_1.0), while also including the latest Boxer assembly (Dog10K_Boxer_Tasha) as an alternate breed. We updated our key farmed animal species including cod, turbot and turkey. For chicken and pig, we released new transcriptomic data tracks for several development timepoints, across a number of tissues as part of the GENE-SWitCH project. These are currently available via Rapid Release and will be included in future annotation updates for the core gene sets on the full Ensembl site.

### Improvements to the regulatory build

We updated our human regulatory build to improve the range of elements annotated on the Y chromosome. The Ensembl regulatory build integrates data from dispersed resources to offer a consistent set of candidate regulatory elements in human and mouse across a diverse range of epigenomes (cell types, cell lines or tissues). It distinguishes different classes of elements: enhancers, promoters, promoter flanking regions, regions of open chromatin and CTCF and TF binding sites. Our human regulatory build identified 622,461 candidate regulatory elements, together with their activity. These were inferred by chromatin marks across 118 epigenomes from Roadmap Epigenomics, ENCODE and BLUEPRINT (20). All primary data sets are available via the International Human Epigenome Consortium (IHEC) (21).

The regulatory build annotation complements other approaches, such as the SCREEN Catalogue, based on open chromatin regions from ENCODE, or emerging approaches using deep learning (22–24). The methods we use to generate the regulatory build will evolve over time as we respond to new data and approaches. Our continued goal is to provide an easily accessible set of regulatory elements integrated with other data in Ensembl.

We now source eQTL data from the eQTL Catalogue (25), which uniformly processes data from a wide range of studies. These are displayed using a Manhattan track in the Regulation section of the Gene view, and the 'Genes and Regulation' section of the Variant view.

### Variant interpretation improvements including data mining scientific publications

To aid genome interpretation across 28 species, we aggregate, annotate and display variation and phenotype association data. The additional new species added this year include variant data for Nile tilapia (EVA accession number: PRJEB38548), American mink (PRJEB26368), great tit (PRJEB24964) and rabbit (PRJEB27278). We pull variants dynamically from the European Variation Archive for these. We extended the catalogues of human allele frequency data we make available, incorporating data from the NCBI Allele Frequency Aggregator (ALFA). ALFA holds summary data for results in the dbGaP database. We also added the GEM Japan Whole Genome Aggregation (GEM-J WGA) Panel, which is derived from the whole genome sequences from 7609 individuals from across Japan.

The scientific literature holds information valuable to variant interpretation. This is not trivial to extract, and different text mining and curation approaches enable access to different information. Mastermind Genomic Search Engine (26) mines full text articles and supplementary information for variants, described as protein or transcript changes. It has over 7.5 million full-text articles and 2.5 million supplemental datasets. We collaborated with Genomenon to release a new track in our browser to improve simple access to information on variants described in the literature. The track can be accessed by selecting the ‘Configure this page’ option from the ‘Region in Detail’ view. This can be found either by typing ‘mastermind’ in the ‘Find a track’ box on the top left, or by opening the ‘Variation’ list and checking the ‘Mastermind variants’ box. The displayed variants link out to detailed reports and publication information on the Mastermind site.

## IMPROVING DATA ACCESS AND TOOLS

### Supporting variation interpretation and analysis

We released a new interface for the Ensembl Variant Recoder (https://www.ensembl.org/Multi/Tools/VR). This is a sister tool to the Ensembl VEP that is designed to translate between different variant names. For example, using a ClinVar (27) identifier such as ‘VCV000018068’, the Ensembl Variant Recoder will return HGVS nomenclature (28), SPDI (29) or other database identifiers including the dbSNP identifier ‘rs699’ (30). It can convert between the many different naming conventions and identifiers used for variants. In addition, it can resolve ambiguous protein-level changes, as often reported in the literature, to multiple alternative alleles. For example, for this input ‘BRCA2:p.Trp31Cys’, the tool will return results for both the T and C alleles. These can be outputted in VCF format, which is handy as it is a format more commonly used as input in other tools.

The Ensembl VEP enables variant annotation, filtering and prioritisation, building on the data available in Ensembl and other resources. We continued to extend the annotations available through VEP. This year we created extensions to integrate variant pathogenicity predictions from ClinPred (31) and PrimateAI (32). We also improved support for MANE transcripts by highlighting MANE Plus Clinical records and now calculate SpliceAI predictions for MANE transcripts. The Ensembl VEP web tool has been updated with links to the Mastermind Genomic Search Engine.

We created a dedicated MANE portal on our Ensembl Transcript Archive (Tark) site, available at http://tark.ensembl.org/web/mane_project/. Tark is our resource for visualisation and comparison of transcripts. It contains current and historical transcript data for transcripts from Ensembl, RefSeq and other sources. We plan to add new features to Tark throughout 2022, expanding the service's functionality and tools, and extending the information held about MANE transcripts.

### Developing a new website

Development of the new Ensembl website (https://2020.ensembl.org) has continued, with significant progress towards this as the replacement for our current infrastructure. This new site has been entirely redesigned with a focus on user journeys. We have conducted extensive user research with different groups to identify common journeys of querying Ensembl. We have used this information to ensure these journeys are more intuitive to accomplish than on our current site. Additionally, the new site adopts more modern web technologies to create a increased responsive experience and to be guided by industry-standard design principles. As a result, these common journeys will become easier and much faster to perform. Here, we describe our progress.

**Figure 2.**
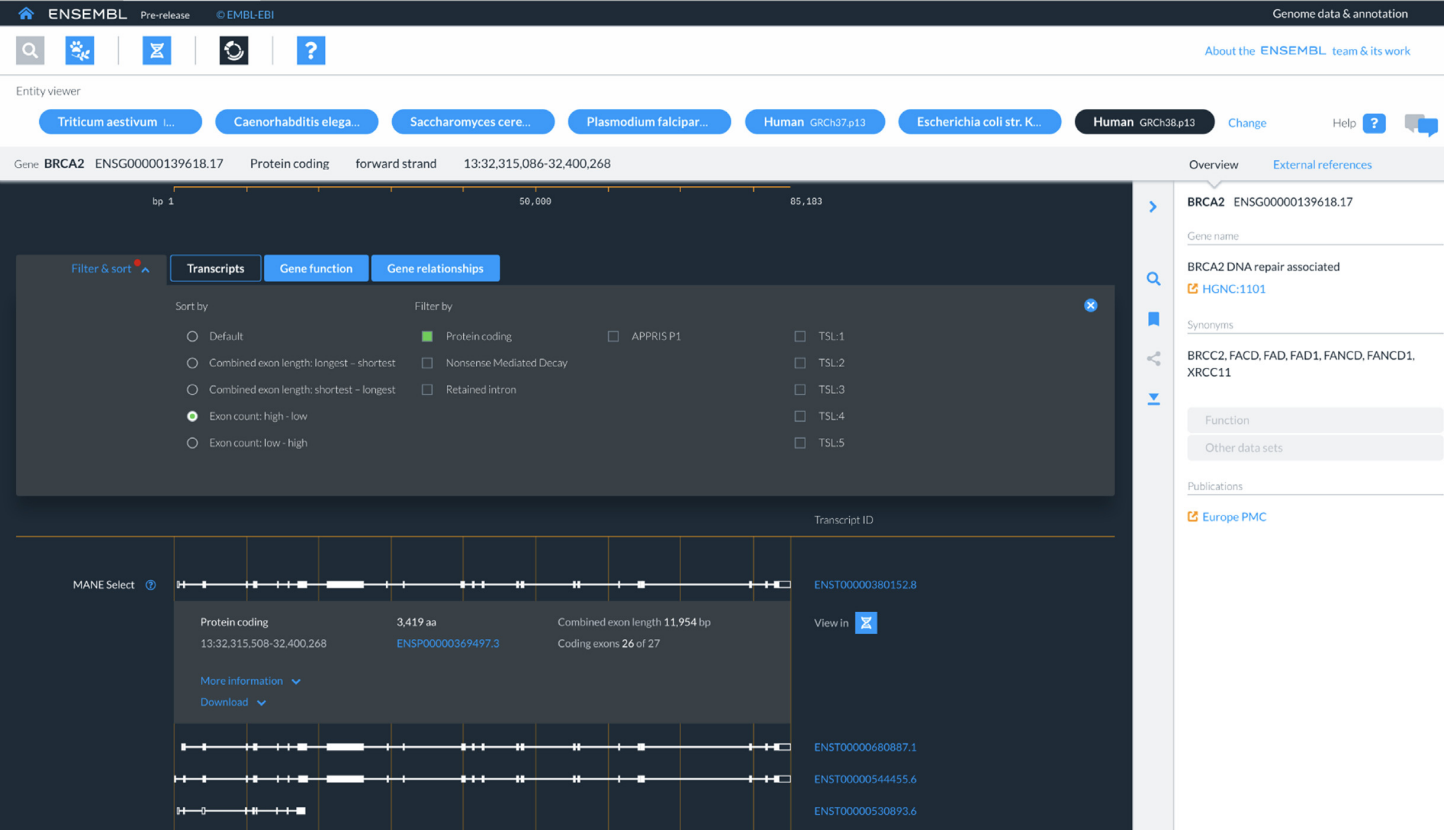
Entity viewer with transcript filtering and sorting. Screenshot of entity viewer showing protein coding transcripts available for BRCA2 (ENSG00000139618.17, GRCh38), ordered according to the number of exons. Information about the selected gene is displayed in the right-hand menu with cross references available by clicking on ‘External references’. Multiple functions are available from the right-hand control strip including a gene search (magnifying glass icon), recently visited genes (bookmark icon) and sequence download (download icon). Additional information about each transcript can be accessed from the ‘More information’ link, and sequence data for a single transcript can be accessed from the ‘Download’ link.

We have deployed a new application, called ‘Entity Viewer’, to inspect properties and linked data related to genes, transcripts and proteins (Figure 2). Entity viewer covers the largest proportion of traffic usage on our full Ensembl current site, and its development is seen as a key component for the new infrastructure. Entity viewer gives access to gene nomenclature symbols, biotypes, and cross references, alongside information about function including differential splicing, protein isoforms, protein domains and 3D structures from PDBe (33). Additional data including orthology, GO annotation, phenotypes, expression, pathways and linked variation are flagged for inclusion. The entity viewer is powered by a GraphQL API, which implements our new data model.

Other major advances include a search application for genes built using Solr, the ability to download sequences of genes and transcripts from a Global Alliance for Genomics and Health standard refget server (34), a help application, pages with information summarising the data associated with each species, and a redesigned home page.

These developments ensure our new site implements a set of basic user journeys that are centred around browsing genomes, inspecting genes and downloading sequences. We continuously assess the new site's suitability via user experience (UX) testing, and use the feedback received to improve these interfaces. Members of the research community are invited to sign up for UX sessions via our helpdesk and help shape our future offerings.

Over the coming year we plan to extend the number of genomes available, provide summary views of variant information, and access to gene orthologues and paralogues. We will enable usage of popular tools including BLAST and Ensembl VEP, and develop an interface to search and download files from our FTP site. Our existing Rapid Release infrastructure will be reused to support data release on the new site ensuring both our current and new infrastructure can release emerging data sets. We believe once these additional annotations are made available, our new infrastructure's functionality will be close to what is available from our current infrastructure but it will be capable of supporting far more complex user journeys and will streamline common journeys.

### User support and training

Despite the challenges of the COVID-19 pandemic, we have continued to deliver help and training for using Ensembl. Our virtual training programme has expanded and now offers training on the Ensembl browser, Ensembl VEP and Ensembl REST API. We prepared and delivered bioinformatics training as part of our work on the AQUA-FAANG consortium https://www.aqua-faang.eu/. These were for EuroFAANG participants https://www.aqua-faang.eu/eurofaang.html and focused on ChIP-seq and ATAC-seq analysis. We also covered general bioinformatics practices such as containerisation as we adopt these for our backend work to update and improve the regulatory build.

We have taken the opportunity to deliver courses with open registration for participants across the globe. We held our usual one-day training courses as a number of shorter sessions, delivering these at different times of day to cater to participants in different time-zones. We also continue to work with host organisations to deliver training to specific groups, tailored to their needs and interests. Details of the training we offer and how to arrange customised courses for your institute can be found at https://training.ensembl.org/. Training courses with open registration are advertised on the Ensembl blog and social media channels including Twitter, Facebook and LinkedIn.

We use several different software platforms to enable virtual training, combining video conferencing platforms, such as Zoom, Webex and Microsoft Teams, with interaction tools, such as living documents (a freely available Google Doc where participants can type questions and answers), Slack and Slido. For technical training on the REST API, we have used the Google Colab cloud environment to distribute Jupyter notebooks without requiring the participants to download or instal anything. The Jupyter notebooks contain live code and narrative text for the participants. For technical training on the Ensembl VEP tool, we used Docker (35), an open-source containerisation platform. This allows us to package VEP into a ‘container’ that contains all the dependencies required to run the code in any environment. This significantly helps the participants access and run the required software. Training materials are all distributed during and after courses using our training site at training.ensembl.org, and video sessions are recorded and hosted on YouTube to send to participants.

After the pandemic, we anticipate a hybrid training model. Some courses will still take place virtually, while travel will resume to deliver other courses in person, following changes in restrictions.

We continue to offer help with specific Ensembl problems on our helpdesk, helpdesk@ensembl.org. Our online courses on EBI Train Online (https://www.ebi.ac.uk/training/on-demand) are kept up-to-date and offer asynchronous virtual training.

## CONCLUSIONS

The annotations created and distributed at no cost by Ensembl—including genes, variants, regulatory elements and phylogenetic trees—serve as reference data that enables scientific discovery. Many researchers also depend on our powerful platform of tools, genome browser and APIs for their data analysis and functional interpretation. Our recent reengineering of the Ensembl annotation and processing methods have transformed the rate at which we can release annotation on new assemblies. This has been vital progress for more global diversity sequencing projects to realise their full potential, supported by timely releases of annotation on a continually increasing number of assemblies.

We remain committed to improving comprehensive genome informatics resources to support the highest quality annotation across reference vertebrate species. We focus on human and mouse in particular. However, as more diverse data types, including haplotypes and tissue-specific data, become available from other important vertebrates, we will adapt and modify our methods and systems to support these significant advances for genomics.

As biological understanding, data availability and genomic complexity have evolved significantly since our last website redesign over a decade ago, we are taking every opportunity to rebuild a modern and flexible new website from the ground up. We are excited to make use of modern programming languages, APIs and new standards to soon deliver an MAP for our new site. We enthusiastically encourage you to test and provide feedback as we continue to develop a fully featured site.

## DATA AVAILABILITY

All Ensembl integrated data are available without restriction from our website (https://www.ensembl.org), in bulk from our FTP site (ftp.ensembl.org) and programmatically via our REST API (https://rest.ensembl.org). Ensembl code is available from GitHub (https://github.com/Ensembl) under an open source Apache 2.0 license. News about our releases and services can be found our blog (https://www.ensembl.info), our announce mailing list (https://lists.ensembl.org/mailman/listinfo/announce), Twitter (@ensembl) and Facebook (https://facebook.com/Ensembl.org). Ensembl and Ensembl VEP are registered trademarks of EMBL.
